# *Drosophila* immune cells extravasate from vessels to wounds using Tre1 GPCR and Rho signaling

**DOI:** 10.1083/jcb.201801013

**Published:** 2018-09-03

**Authors:** Leila Thuma, Deborah Carter, Helen Weavers, Paul Martin

**Affiliations:** 1 Department of Physiology, Pharmacology and Neuroscience, Biomedical Sciences, University of Bristol, Bristol, UK; 2 School of Cellular and Molecular Medicine, Biomedical Sciences, University of Bristol, Bristol, UK; 3 School of Biochemistry, Biomedical Sciences, University of Bristol, Bristol, UK; 4 School of Medicine, Cardiff University, Cardiff, UK

## Abstract

Inflammation is pivotal to fight infection, clear debris, and orchestrate repair of injured tissues. Although *Drosophila melanogaster* have proven invaluable for studying extravascular recruitment of innate immune cells (hemocytes) to wounds, they have been somewhat neglected as viable models to investigate a key rate-limiting component of inflammation—that of immune cell extravasation across vessel walls—due to their open circulation. We have now identified a period during pupal development when wing hearts pulse hemolymph, including circulating hemocytes, through developing wing veins. Wounding near these vessels triggers local immune cell extravasation, enabling live imaging and correlative light-electron microscopy of these events in vivo. We show that RNAi knockdown of immune cell integrin blocks diapedesis, just as in vertebrates, and we uncover a novel role for Rho-like signaling through the GPCR Tre1, a gene previously implicated in the trans-epithelial migration of germ cells. We believe this new *Drosophila* model complements current murine models and provides new mechanistic insight into immune cell extravasation.

## Abstract (precis)

In contrast to vertebrates, adult *Drosophila melanogaster* have an open cardiovascular system. However, Thuma et al. find that in late pupation, hemolymph flows through *Drosophila* wing veins, providing a unique genetic and live-imaging opportunity to investigate the mechanisms driving immune cell extravasation from vessels to wounds and reveal new roles for Tre1 and Rho signaling in this process.

## Introduction

Following damage to, or infection of, vertebrate tissues, circulating leukocytes must be recruited to the wound site from the circulation. Murine genetic approaches, combined with complementary in vitro flow studies, have identified a series of key overlapping steps in this process, termed the “leukocyte-adhesion cascade” (Ley et al., 2007). Leukocytes are first captured by adhesion to the walls of post-capillary venules in the vicinity of the insult and subsequently extravasate through the cell and matrix layers of the vessel wall before extravascularly migrating the remaining short distance to the site of damage (Kolaczkowska and Kubes, 2013; Nourshargh and Alon, 2014). Leukocyte transmigration through the vessel wall (“diapedesis”) involves a coordinated series of complex molecular and morphological changes in both immune cells and endothelial cells lining that local region of the venule (Voisin and Nourshargh, 2013; Muller, 2015, 2016; Vestweber, 2015). A similar process occurs when circulating cancer cells leave the vasculature to establish a secondary tumor site and is a rate-limiting component in the metastatic spread of cancer (Madsen and Sahai, 2010; Reymond et al., 2013).

Advances in multi-photon imaging, together with sophisticated 4D image analysis software, have now made it possible to follow leukocyte extravasation live, in vivo, using confocal intravital microscopy in some regions of the body (Mempel et al., 2004; Voisin et al., 2009; Woodfin et al., 2011; Kolaczkowska and Kubes, 2013; Colom et al., 2015). Transmission electron microscopy (TEM) studies have also provided new ultrastructural insights into the morphological changes within extravasating leukocytes and endothelial cells (Marchesi and Florey, 1960; Hurley, 1963; Nourshargh et al., 2010), although it has proven difficult to directly correlate precise moments of these ultrastructural events with in vivo imaging observations. While the identities of many key molecular and cellular players in leukocyte extravasation are well established, there still remains a clear need for better understanding of the precise signaling dynamics, cellular interactions, and phenotypic changes that occur during this complex immune response.

*Drosophila melanogaster* has become a valuable system in which to dissect fundamental and conserved aspects of the inflammatory wound response due to its genetic tractability (permitting precise spatio-temporal genetic manipulation) combined with optical translucency for high-resolution in vivo imaging (Razzell et al., 2011; Wood and Martin, 2017). However, *Drosophila* possess an “open” circulatory system without discrete blood vessels and have been largely overlooked as viable models to investigate immune cell diapedesis. While proxy models have been developed, including “clot” capture of hemocytes in larval wounds (Babcock et al., 2008) and hemocyte invasion into the tail epithelium during embryonic development (Siekhaus et al., 2010), the majority of studies of the wound inflammatory response in *Drosophila* have modeled the “extravascular” phase of leukocyte recruitment through the interstitium to sites of damage (Stramer et al., 2005; Moreira et al., 2010; Weavers et al., 2016a,b). However, in this study, we identify a period during *Drosophila* pupal development when beating wing hearts pulse blood (including circulating immune cells) through developing wing veins. Strikingly, wounding the epithelium adjacent to these vessels triggers a local inflammatory response, enabling live imaging of immune cell extravasation events in real time at high spatio-temporal resolution in vivo—and offering the potential for further detailed genetic dissection of immune cell extravasation from vessels to damaged tissue within *Drosophila*.

## Results and discussion

### *Drosophila* pupal wing veins carry circulating immune cells

**Figure 1.**
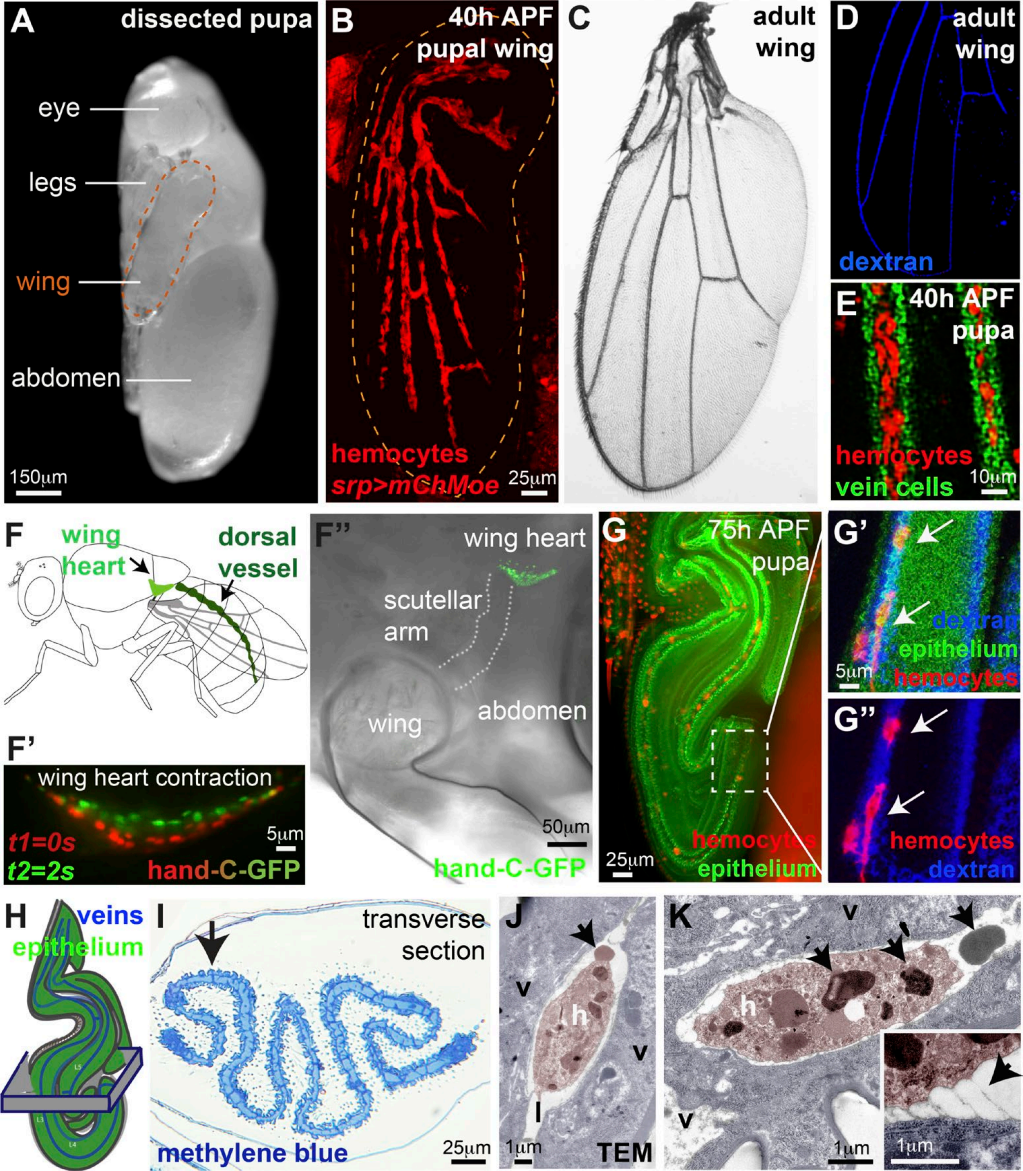
***Drosophila* pupal wing veins carry circulating hemocytes. (A)** 75-h APF *Drosophila* pupa dissected from pupal case for imaging. **(B­–D)** Hemocytes (red, *srp>moesin-mCherry,* B) restricted to wing veins by 40 h APF (B) in a pattern reminiscent of adult vessels (C, brightfield, and D, dextran-loaded veins, blue). **(E)** Hemocytes (red) within lumens of 40-h APF wing veins (vein wall cells labeled using *shortvein>GFP*, green). **(F)** Wing hearts (F) contract (F’ and F’’, hand-C-GFP reporter) to pump hemolymph into the veins. **(G)** 75-h APF wings are folded (ubiquitous Moesin-GFP, green), but hemocytes (arrows, red, *srp>mch-moesin*) remain within vein lumens (G’ and G’’, blue dextran). **(H and I)** Folded nature of wing revealed by resin histology (H, schematic, and I, methylene blue; arrow in I indicates flat epithelium adjacent to vein). **(J and K)** Hemocytes (h, false-colored pink in transmission electron micrograph, TEM) within the lumen (labeled l) of 75-h APF wing veins (v, vein wall cells, false-colored blue) contain large cytoplasmic granules (arrows, K) and are occasionally tethered to the vein wall (arrow, inset K). Also see Fig. S1 and Videos 1, 2, 3, 4, and 5.

*Drosophila* pupae have recently been established as powerful in vivo models to study the extravascular recruitment of innate immune cells (hemocytes) to wounds (Sander et al., 2013; Weavers et al., 2016b). To determine whether there might be a suitable stage during *Drosophila* pupal development to model immune cell diapedesis, we examined the distribution of hemocytes throughout wing morphogenesis. Strikingly, once the pupal wing epithelia have fused together to form the adult-like pattern of wing veins at 40 h after puparium formation (APF; Fristrom et al., 1993; de Celis, 2003), most hemocytes become restricted to the lumens of these wing veins (Fig. 1, A–D; Fig. S1, A and B; and Video 1), which are enclosed by specialized vein wall cells (Fig. 1 E; Fristrom et al., 1993; Sotillos and De Celis, 2005; Molnar et al., 2006). In rare cases, individual hemocytes remain outside the developing wing veins (data not shown). Dextran loading of the pupal wing veins reveals how their pattern is a miniature topological model of the adult wing vein pattern (Fig. S1, A and B; and Video 1). Contractile wing hearts (Fig. 1, F–F’’) are associated with each pupal wing and establish a pulsatile flow of hemolymph through the narrow wing veins (Tögel et al., 2008, 2013). By 75 h APF, when the wing hearts began pulsating (Fig. 1 F’), we observed injected fluorescent beads moving through wing veins with speeds of up to 25 µm/min (mean of 15.2 µm/min, SD = 5.9, *n* = 16; Fig. S1, C and D). Such rapid flow was also apparent in adult wing veins (data not shown) when we observed hemolymph (together with individual hemocytes) moving at even faster rates (mean of 6.45 µm/s, SD = 2.1).

**Figure 2.**
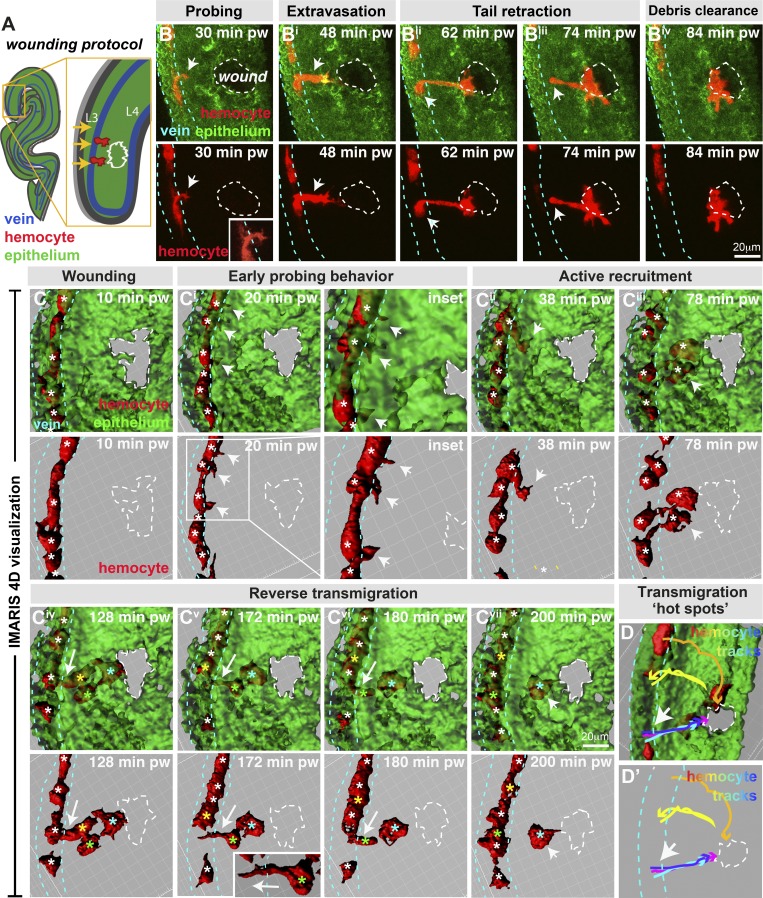
***Drosophila* wing vein hemocytes extravasate from vessels to wounds. (A and B)** Wounding of pupal wing epithelium (green, ubiquitous GFP-Moesin; schematic, A, and in vivo imaging, B) triggers hemocyte (red, *srp>mch-moesin*) extravasation from wing veins (blue dashed lines, position determined from z-sections) to sites of damage (white dashed lines). **(C)** IMARIS software permits 3D visualization of imaging data. Hemocytes (red) probe the vessel wall (arrows, B and insets in B and Ci) and extravasate from the vessel (arrows, Bi–Biii and Cii), requiring retraction of the hemocyte tail (arrow, Biii). Multiple hemocytes exit the vessel (Civ), but some return to the vessel (arrows, Civ–Cvii; individual hemocytes indicated by asterisks: white, all hemocytes in C–Ciii and hemocytes in vessels, Civ–Cvii; cyan, extravasated hemocytes that remain at the wound; yellow and green, reverse migrating hemocytes). **(D)** Tracking hemocyte trajectories indicates multiple hemocytes extravasating from similar vein wall locations (arrows; blue, cyan, and magenta tracks), although hemocytes also exit from additional locations (yellow and orange tracks). pw, post-wounding. Also see Videos 6 and 7.

By 75 h APF, the pupal wing had become highly folded, and the veins followed a convoluted path (Fig. 1 G, Video 2, and Video 3). High-resolution imaging of the flattest, distal-most wing region reveals a continuous stretch of wing vein (labeled by fluorescent dextran, Fig. 1, G’ and G’’) in which hemocytes remain amenable to high-resolution in vivo imaging (Video 4) for up to 20 h until the time of pupal hatching. Despite the folded nature of the wing (Fig. 1, H and I), an area of intervein epithelium remains flat (arrow, Fig. 1 I) adjacent to vein L3, making it particularly suitable for wounding experiments (see Fig. 2). TEM of this region reveals a number of ultrastructural details of hemocytes and their relationship with the vessel wall (Fig. 1, J and K; and Fig. S1 E). Hemocytes are densely packed with cytoplasmic granules that may be akin to neutrophil granules containing antibacterial and proteolytic proteins (arrows, Fig. 1, J and K; Borregaard et al., 2007). Our TEM studies also show occasional direct contacts or “tethers” of hemocytes with the vessel wall (inset, Fig. 1 K; *n* = 3), suggesting that hemocytes are not always free-flowing within the lumen of the vein, despite a small but significant increase in hemocyte speed from 27 h APF to 75 h APF following wing heart maturation (Fig. S1 F). Indeed, our live-imaging observations of hemocyte vascular patrolling occasionally showed hemocytes even migrating against the flow of hemolymph (Videos 4 and 5). The same is true in vertebrates, where, even in noninflammatory settings, macrophages have been shown to adhere to and patrol the luminal surface of vessels, sometimes moving against the flow of blood (Auffray et al., 2007; Carlin et al., 2013). Unlike the classical apical (luminal) and basal (abluminal) polarity of vertebrate endothelial cells (Charpentier and Conlon, 2014), *Drosophila* wing vein cells face the lumen with their basal side (Fristrom et al., 1993; O’Keefe et al., 2007). Intriguingly, *Drosophila* vein cells display atypical localization of certain classical basal markers, such as β-integrin, which is absent from their basal (luminal) surface (Fristrom et al., 1993).

### *Drosophila* immune cells extravasate from wing veins to sites of tissue damage

Using the distal-most region of 75-h APF pupal wings, we generated small 40-µm-diameter laser wounds within the wing epithelium at ∼40 µm from vein L3 (Fig. 2, A and B). Strikingly, this injury reproducibly triggered local hemocyte extravasation from the vessels into the damaged tissue (Fig. 2 B and Video 6). Within 30 min of injury, hemocytes locally halted their flow and began interacting with the vessel wall, extending pseudopodial-like probes along and through the luminal surface as though scanning for optimal exit sites (Fig. 2, B and E; and Video 6; mean 48.7 ± 17% of hemocytes passing the wound site, *n* > 300, and 77% of videos examined, *n* > 30). Similar probing behavior is highly characteristic of vertebrate leukocytes that extend protrusions through junctions between adjacent endothelial cells or into the endothelial cell body in order to detect chemotactic gradients associated with the endothelium or subendothelial space (Carman et al., 2007; Nourshargh and Alon, 2014). In the majority of wounds examined (65% of videos, *n* > 30, up to 6 h after injury), hemocyte probing led to at least one hemocyte successfully extravasating through the vessel wall and migrating across the extravascular space to the site of damage (Fig. 2 B). Extravasating hemocytes underwent a series of dramatic morphological changes during vessel exit, generating a dynamic protrusive leading edge in the direction of migration and a rear contractile uropod during tail retraction (arrows, Fig. 2 B).

Using specialist IMARIS software, which offers superior spatial resolution than traditional z-stack maximum intensity projections, we visualized the 4D time-lapse imaging data as dynamic 3D videos (Fig. 2 C and Video 7). By 2 h after wounding, multiple hemocytes had extravasated from the vessel (on average 10.3% of hemocytes passing the wound site, *n* > 300), and on average 70.2% (*n* > 35) of these extravasated hemocytes reached the injury site (Fig. 2 Civ; mean of 2.1 hemocytes, SD = 1.1, *n* = 29 videos), where they often remained for several hours phagocytosing necrotic wound debris (Fig. 2 Cvii) until the wound had reepithelialized. However, occasional emigrated hemocytes (34.2% on average, *n* > 25) reverse transmigrated back toward, and into, the vessel (Fig. 2, Civ–Cvi; see color-coded asterisks of reverse migrating hemocytes), often at the same site from which they first emerged, as previously reported for neutrophil reverse migration in zebrafish and mice (Mathias et al., 2006; Tauzin et al., 2014; Colom et al., 2015; Ellett et al., 2015; Nourshargh et al., 2016; Wang et al., 2017). While the majority of extravasation is complete by 2 h after wounding, long-term imaging revealed that hemocytes could be recruited for up to 10 h after the completion of wound closure (data not shown). Intriguingly, hemocyte trajectory analysis suggests the existence of particular “hot spots” of extravasation, with the majority of hemocytes leaving the vessel immediately adjacent to the wound and several hemocytes streaming out, one after another (Fig. 2, D and D’), although hemocytes could also extravasate from the vessel up to 100 µm up- or downstream of the wound. Following extravasation, hemocytes migrated with a mean velocity of 3.8 µm/min (SD = 0.7) through the extravascular space to the injury site, similar to our observations for hemocytes migrating to pupal wounds at stages before vessel formation (Weavers et al., 2016b). We observed only rarely hemocytes in this wing vessel region (Fig. 1 I) extravasating from the vein in unwounded conditions (in 6.7% of videos examined, a single hemocyte extravasated, *n* = 30), perhaps triggered by local apoptosis in the intervein epithelium. Although these extravascular hemocytes are responsive to epithelial wounds (data not shown), they were excluded from our quantification if they had extravasated before wounding.

### Correlative light-electron microscopy (CLEM) captures specific ultrastructural details during hemocyte extravasation

**Figure 3.**
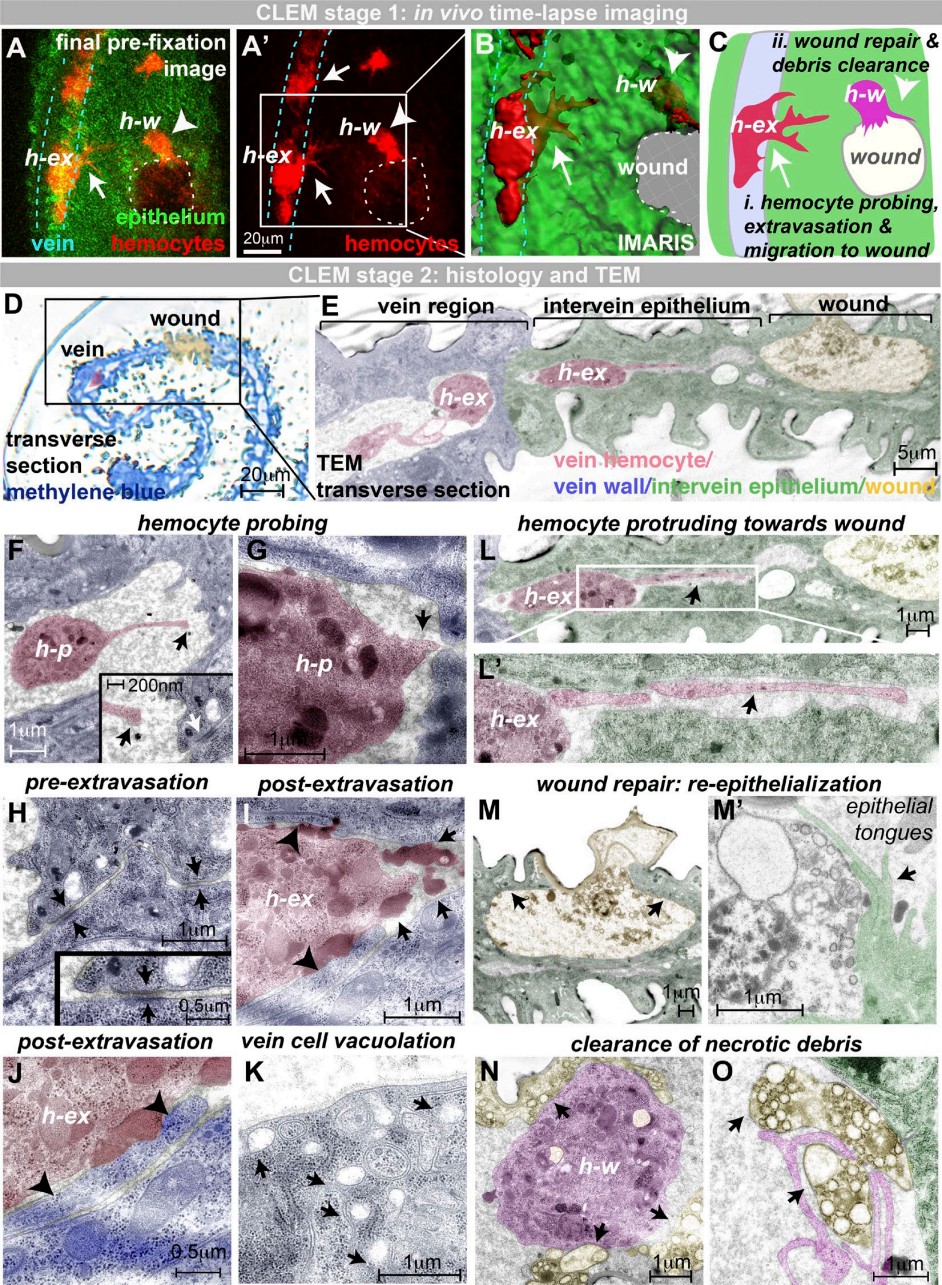
**CLEM of hemocyte extravasation. (A)** In vivo imaging of extravasation (hemocytes, red, *srp>mch-moesin;* and epithelium, green, ubiquitous GFP-Moesin). **(A–C)** Samples fixed at key moments (hemocyte, h-ex, initiating transmigration; and hemocyte, h-w, reached the wound). Arrows indicate hemocytes in the process of extravasation (h-ex), and arrowheads indicate extravasated hemocytes at wound (h-w). **(D–O)** Samples prepared for TEM. **(D and E)** Resin transverse section (D, methylene blue) and TEM section (E) illustrate the route from vein to wound with a hemocyte (h-ex, false-colored red) traversing the vein wall (E). **(F and G)** TEM reveals hemocyte probing of the vessel wall, with long (F) and short (G) protrusions targeting junctions between adjacent vein wall cells (arrow, G). Arrows (black, F) indicate the end of the long hemocyte probing protrusion. White arrow (F, inset) indicates intact vein wall junction. **(H–K)** Prior to extravasation, the vein wall barrier is intact (H; arrows, vein wall junctions), but extravasating hemocytes move across and flatten this vein wall barrier (arrowheads, I and J). Extravasating hemocytes are associated with large darkly staining deposits (arrows, I), and vein cells exhibit vacuolation (arrows, K). **(L–O)** Hemocytes use long protrusions to migrate through the extravascular space toward the wound (arrow, L). Wounds are repaired by long epithelial tongues (arrows, M and M'). Hemocytes at the wound site (magenta, N and O) clear up necrotic wound debris (yellow, indicated by arrows). All images were taken from two 75-h APF *Drosophila* pupae prepared for CLEM. Also see Fig. S2.

Live imaging of wounded pupal wings allowed us to pinpoint precise moments when hemocytes extravasate from vessels (Fig. 2). To capture ultrastructural details of these events, we fixed individual pupae at key moments to enable CLEM, a technique previously used to analyze in vitro intracellular trafficking (de Boer et al., 2015). In this way, we captured single time points that enable us to study (i) hemocytes in the act of extravasation (h-ex; Fig. 3, A–C and F–J); (ii) extravasated hemocytes en route to the wound (Fig. 3 L); as well as (iii) hemocytes at the wound actively clearing cell debris (h-w; Fig. 3, A–C, N, and O). Low-magnification transverse sections demonstrate the route of hemocyte extravasation from the vein lumen, through the interstitium to the site of tissue damage (resin histology, Fig. 3 D, and TEM, Fig. 3 E), while high-magnification serial TEM sections reveal cellular through to ultrastructural details of hemocyte extravasation (Fig. 3, F–K; and Fig. S2, A and B) and post-extravasation migration toward the wound (Fig. 3, L–O; and Fig. S2, E–H).

Extravasating hemocytes (h-p) extend probing protrusions toward the vein wall (Fig. 3 F) that target the intercellular junctions between adjacent vein wall cells (arrow, Fig. 3 G). *Drosophila* vein wall cells are linked by tight intercellular adherens junctions (Fig. 3 H and Fig. S2, A and A’; O’Keefe et al., 2007), as in vertebrate vessels (Muller, 2003), but, at sites of hemocyte extravasation, we observed dramatic ultrastructural changes (Fig. 3, I and J; and Fig. S2 B). Vein wall cell contacts are interrupted (Fig. 3 I and Fig. S2 B), and we observed long vein cell “tongues” (arrowheads, Fig. 3, I and J), suggesting that the vein cell barrier might have been flattened by extravasating hemocytes; this is consistent with live-imaging observations of dextran leakage from the wing veins following wounding (Fig. S2 C). Loosening of junctions is also characteristic of vertebrate immune cell extravasation (Muller, 2011), as vertebrate immune cells generate gaps of micron-scale size when squeezing through inflamed endothelial barriers in vitro and in vivo (Shulman et al., 2009). Extravasating hemocytes are closely associated with numerous darkly staining dense deposits (arrows, Fig. 3 I; and Fig. S2 D) that were also observed inside (and in the vicinity of) hemocytes in unwounded conditions (Fig. 1, J and K), which we speculate might reflect mechanisms that facilitate exit from the vein. The vein cells themselves became highly vacuolated (Fig. 3 K), reminiscent of the vacuoles observed in endothelial cells during vertebrate immune cell diapedesis (Muller, 2015, 2016).

Following extravasation, hemocytes extend long leading-edge protrusions through small gaps (arrows, Fig. 3, L and L’) between upper and lower intervein epithelia as they migrate toward the wound and exhibit slender retracting uropods at their rear (Fig. S2 F). This matrix-rich, extracellular space between intervein epithelia is present even in unwounded tissue (Fig. S2 E; Fristrom et al., 1993). Less organized patches of extracellular material were observed in advance of extravasated hemocytes (Fig. S2 G), suggesting that hemocytes might remodel material in the interstitial space as they migrate toward the wound. Similar material is sometimes observed within extravasated hemocytes (Fig. S2 H). Interestingly, we found that levels of the secreted Matrix Metalloproteinase 1 (MMP1; Stevens and Page-McCaw, 2012) increased following wounding (Fig. S2, I–K), and we observed the appearance of punctae of Collagen IV (*Drosophila* Viking) within hemocytes (Fig. S2 L), although it is unclear whether this reflects hemocyte synthesis or internalization of Collagen IV. Epithelial wound repair occurs in parallel with the inflammatory response, and we observed wound edge epithelial tongues extending toward one another to seal the wound gap (Fig. 3 M). On reaching the wound, hemocytes play an active role in the repair process by phagocytic clearance of necrotic debris (arrows, Fig. 3, N and O).

### *Drosophila* hemocyte extravasation from veins is integrin dependent

**Figure 4.**
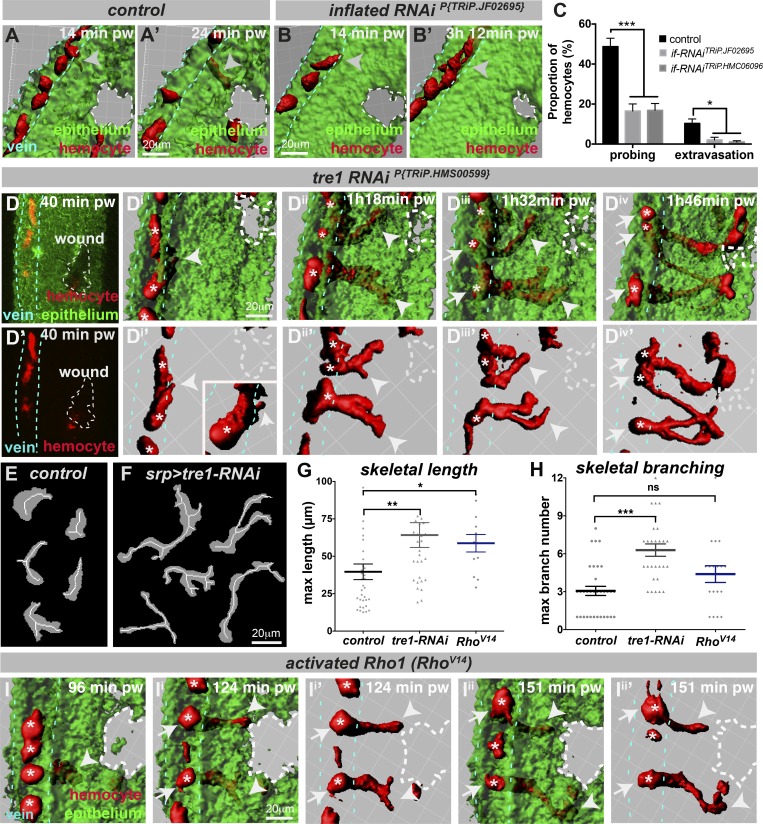
**Hemocyte extravasation requires integrins and the GPCR Tre1. (A–C)** IMARIS visualization of control (A) and hemocyte-specific *inflated-RNAi^P{TRiP.JF02695}^* hemocytes (B, using *srp-Gal4*) in 75-h APF pupal wings. Unlike controls (red, arrowheads, A and A’), hemocytes with *inflated-RNAi^P{TRiP.JF02695}^* exhibit defects in vessel extravasation (arrowheads, B and B’, and quantified in C). **(D–H)** Hemocytes with *tre1-RNAi^P{TRiP.HMS00599}^* (using *srp-Gal4*) become significantly elongated and branched during extravasation (arrowheads, Dii and Diii, and morphology quantification, E–H) and fail to retract their vessel-bound tails (arrows, Div). Arrowhead in Di indicates hemocyte probing of the vessel wall. Arrows in Diii indicate hemocyte tails that are retained within the lumen of the vessel. **(I)** Hemocytes with activated Rho1 (*srp>Rho^V14^*) exhibit similar extravasation defects, becoming significantly elongated (G and arrowheads, I) and failing to retract their tails from the vessel (arrows, I). Asterisks (white) indicate individual hemocytes. **(C, G, and H)** Data are represented as mean ± SEM; ns, not significant; *P < 0.05, **P < 0.01, and ***P < 0.001 via multiple *t* tests (C) or one-way ANOVA (G and H), both with Sidak’s correction for multiple comparisons. pw, post-wounding. Also see Fig. S3 and Videos 8 and 9.

The arrest of leukocytes on the luminal endothelium surface at the site of vertebrate inflammation requires activation of at least one of the major leukocyte integrins (Dustin and Springer, 1988; Elices et al., 1990; Hynes, 1992; Schenkel et al., 2004; Phillipson et al., 2006; Herter and Zarbock, 2013). We therefore tested whether integrins play a conserved role during *Drosophila* immune cell extravasation. *Drosophila* possess five integrin α-subunits (αPS1–5) and two integrin β-subunits (βPS and βν; Bulgakova et al., 2012); the αPS2 integrin *inflated* is known to be required for the invasive developmental migration of embryonic hemocytes into the tail (Siekhaus et al., 2010) and early pupal hemocyte motility (Moreira et al., 2013). We found that RNAi-mediated knockdown of *inflated* integrin (using multiple independent RNAi lines) within late pupal hemocytes (using the hemocyte-specific *srp-Gal4* driver) caused dramatic defects in hemocyte extravasation (Fig. 4, A–C; Fig. S3, A and B; and Video 8). *inflated-RNAi* hemocytes rarely arrest and extend probing protrusions across the vessel wall adjacent to the injured tissue (Fig. 4 C and Fig. S3 A; imaging for 3 h after injury). Consequently, significantly fewer *inflated-RNAi* hemocytes successfully extravasate from the vessels (Fig. 4 C; extravasation observed in 6.7% or 13.3% of videos for *inflated-RNAi TRiP.JF02695* or *TRiP.HMC06096*, respectively, versus 38% of controls over short-term 3-h imaging). We saw no significant defect in the movement of hemocytes within pupal wing veins before (or after) wounding following *inflated-RNAi* (Fig. S3 B), suggesting that basal hemocyte motility within veins of 75-h AFP pupal wings does not require *inflated*. Hemocytes lacking *inflated*, however, migrated more slowly toward the wound following extravasation, suggesting that post-extravasation migration does require *inflated* (Fig. S3 B).

### Rho-like signaling through the G protein–coupled receptor (GPCR) Tre1 is required for hemocyte extravasation

Since wound repair often recapitulates earlier developmental processes (Martin and Parkhurst, 2004), we speculated whether mechanisms used by migrating germ cells to navigate across the gaps within the developing midgut epithelium (Richardson and Lehmann, 2010; Seifert and Lehmann, 2012) might also operate during immune cell diapedesis. One GPCR of the Rhodopsin family known to play a key role in germ cell transepithelial migration is *Drosophila* Tre1 (Trapped in endoderm 1; Kunwar et al., 2003, 2008). Given that *tre1* is expressed in *Drosophila* hemocytes (Fig. S3 C) and its orthologue in mammals, GPR84, is also highly expressed in macrophages (Lattin et al., 2008), the function(s) of Tre1 might be shared between germ cells and innate immune cells, although its role during vertebrate leukocyte diapedesis has not yet been examined.

To test for a role for Tre1 during *Drosophila* immune cell extravasation, we performed hemocyte-specific *tre1-RNAi* (using multiple RNAi lines) and observed a dramatic effect on hemocyte behavior (Fig. 4 D; Fig. S3, D–K; and Video 9). Although *tre1-RNAi* hemocytes migrated at normal speeds within the veins both before and after wounding (Fig. S3 D), in contrast to the normal protrusive behavior of control hemocytes (Fig. 2 and Fig. 4 A), hemocytes lacking Tre1 became highly elongated and branched as they probed and attempted to exit the vessel (Fig. 4, D–H; and Fig. S3, E–K). Successful RNAi-mediated knockdown of *tre1* was confirmed by quantitative RT-PCR (Fig. S3 E). Quantification of hemocyte morphologies using customized image analysis software (Fig. 4, E–H; and Fig. S3, H–J) confirmed that *tre1-RNAi* hemocytes become significantly elongated (Fig. 4 G and Fig. S3 J) and branched (Fig. 4 H) during extravasation. These elongated hemocytes have difficulty releasing their tails from the vessel and retain long tethers linking them to the inside of the vein (arrows, Fig. 4, Diii and Div), even after the leading edge has reached the wound (Fig. 4 D). While control hemocytes briefly probed and completed transmigration in 22 min (*n* > 20, Fig. S3 K), *tre1-RNAi* hemocytes took significantly longer, with those that eventually left the vessel taking 51 min or 44 min (for TRiP.HMS00599 or VDRC#7220 RNAi lines, respectively, *n* > 10) and with the rest fruitlessly attempting to leave the vessel wall throughout the remaining imaging period (Fig. S3 K). Consequently, *tre1*-*RNAi* hemocytes are less efficient at completing extravasation compared with controls (with only 22% of extravasation attempts by *tre1*-*RNAi^(TRiP.HMS00599)^* hemocytes leading to successful exit, compared with 31% of control attempts). Crucially, the requirement for Tre1 in pupal hemocytes is specific for extravasation, since *tre1-RNAi* hemocytes migrate normally to laser-induced wounds in earlier 18-h pupal wings at stages before vein formation (Fig. S3, L–N). This suggests that the dramatically elongated and branched phenotype of hemocytes lacking Tre1 is a direct consequence of attempting extravasation.

Thus, yet again, wound repair recapitulates development with our finding that Tre1, a GPCR essential for embryonic germ cell migration, also regulates immune cell extravasation to sites of damage. The phenotype of *tre1-RNAi* hemocytes resembles that of migrating vertebrate and *Drosophila* immune cells that are defective in Rho GTPase signaling (Ridley et al., 2003; Stramer et al., 2005). RhoA is required within vertebrate immune cells for myosin-based contraction of the trailing edge during transendothelial migration (Worthylake et al., 2001). To test whether Rho function is conserved during *Drosophila* hemocyte extravasation, we manipulated Rho1 activity using dominant-negative (Rho^N19^) and constitutively active (Rho^V14^) constructs. While hemocytes with inactivated Rho took significantly longer to extravasate from vessels (Fig. S3 K), hemocytes with constitutively active Rho became highly elongated as they continued to probe and fruitlessly attempt extravasation, unable to retract their vessel-bound tails, similar to that seen following *tre1-RNAi* (Fig. 4, G and I; and Fig. S3 K), although the effect on branching was less pronounced (Fig. 4 H). Interestingly, unlike loss of *inflated* or *tre1*, manipulation of hemocyte Rho1 caused a significant reduction in hemocyte migration speed within the vein, even in unwounded conditions (3.2 ± 1.2 µm/min for Rho^N19^ and 2.8 ± 1.8 µm/min for Rho^V14^ versus 4.1 ± 1.3 µm/min for controls, P < 0.01).

These data suggest that Rho activity must be dynamically and tightly regulated during hemocyte extravasation. Intriguingly, Tre-1 regulates Rho1 localization within transmigrating germ cells (Kunwar et al., 2008), and recent work has uncovered a conserved protein domain within Tre1, the NPIIY motif, which is required for Rho1-dependent germ cell repolarization (LeBlanc and Lehmann, 2017); whether Tre1 directly affects Rho localization or activity within extravasating hemocytes remains unclear. Interestingly, the mammalian Tre1 homologue, GPR84, is a medium chain fatty acid–sensing GPCR (Wang et al., 2006), and a second conserved (NRY) domain within *Drosophila* Tre1 acts in concert with the lipid phosphate phosphatase Wunen to promote germ cell migration after midgut exit (LeBlanc and Lehmann, 2017). Given that the identities of the attractant cues that draw immune cells from vessels to the site of damage remain unclear, the parallels we observed between immune cells and germ cells may offer useful insights here too.

*Drosophila* offers the opportunity to perform large-scale genome-wide screens to identify novel players required for immune cell extravasation, as hundreds of candidate genes can be knocked out within immune or vein cell lineages using RNAi libraries (Mohr et al., 2014). To achieve more precise knockdown within hemocytes at specific pupal stages, hemocyte drivers such as *srp-Gal4* (Brückner et al., 2004) or *hml-Gal4* (Goto et al., 2003; late embryonic stages onward) could be used in conjunction with a temperature-sensitive Gal80 construct to temporally restrict Gal4 activity (del Valle Rodríguez et al., 2012). By exploiting this genetic tractability, together with potential for high-resolution in vivo imaging, our new *Drosophila* model of immune cell extravasation offers exciting opportunities for unraveling conserved mechanisms underpinning leukocyte diapedesis. Vertebrate leukocytes exhibit an altered phenotype, enhanced survival, and increased effector functions following transmigration (Nourshargh et al., 2010; Stark et al., 2013). Given our recent work showing that *Drosophila* immune cells are primed by environmental cues (Weavers et al., 2016a), our *Drosophila* model could help dissect the molecular mechanisms underlying transmigration-induced priming. There will inevitably be many differences between *Drosophila* immune cell extravasation and the analogous episodes that occur as immune cells (and cancer cells) leave vertebrate vessels, including differences at the level of vessels such as the absence of a pericyte layer. Nevertheless, we have already revealed clear parallels, and the unique opportunities for live imaging and genetic dissection, and potential for screening in flies, are likely to offer complementary insights to those gained from mammalian studies alone.

## Materials and methods

### *Drosophila* stocks and genetics

Fly stocks were maintained according to standard protocols (Greenspan, 1997). The following *Drosophila* stocks were used: *ubiquitous-Moesin-GFP*, *serpent-Gal4* (hemocyte-specific driver; Brückner et al., 2004), *UAS-mCherry-Moesin*, *UAS-GFP*, *ubi-Ecad-GFP*, *Neuroglian-GFP*, *UAS-nuclearRFP* (“red-stinger”), Collagen IV protein trap *viking-GFP* (Morin et al., 2001), *shv-Gal4* (gift from José Félix de Celis, Centro de Biología Molecular Severo Ochoa, Madrid, Spain), *UAS-tre1-RNAi* lines P{TRiP.HMS00599} (Bloomington Drosophila Stock Center #33718) and P{GD715} (VDRC #7220), *UAS-inflated-RNAi* lines P{TRiP.JF02695} (Bloomington #27544) and P{TRiP.HMC06096} (Bloomington #65346), *hand-C-GFP* (Sellin et al., 2006), *UAS-Rho^N19^*, and *UAS-Rho^V14^*. *Drosophila* mutants and transgenic lines were obtained from the Bloomington Drosophila Stock Center unless otherwise stated. The following precise genotypes were used in individual figure panels: *;ubi-Moesin-GFP, srp-Gal4>UAS-mCherry-Moesin;* (Fig. 1, A–D and G–K; Fig. S1 E; Fig. 2; Fig. 3; and Fig. S2, A–K), *;shv-Gal4>UAS-GFP;srp-Gal4>UAS-nRFP* (Fig. 1 E), *hand-C-GFP* (Fig. 1 F), *srp-Gal4>UAS-nRFP* (Fig. S1 B), *srp-Gal4>UAS-mCherry-Moesin* (Fig. S1, C, D, and F), *viking-GFP, srp-Gal4>UAS-mCherry-Moesin* (Fig. S2 L), *ubi-Moesin-GFP, srp-Gal4>UAS-mCherry-Moesin/+* (controls in Fig. 4 and Fig. S3, D and E), *ubi-Moesin-GFP,srp-Gal4>UAS-mCherry-Moesin/+;UAS-if-RNAi.P{TRiP.JF02695}/+* (Fig. 4, B and C; and Fig. S3 B), *ubi-Moesin-GFP,srp-Gal4>UAS-mCherry-Moesin/UAS-if-RNAi.P{TRiP.HMC06096}* (Fig. 4 C and Fig. S3, A and B), *ubi-Moesin-GFP,srp-Gal4>UAS-mCherry-Moesin/+;UAS-tre1-RNAi.P{TRiP.HMS00599}/+* (Fig. 4, D–H; and Fig. S3, D–F and K–N), *ubi-Moesin-GFP,srp-Gal4>UAS-mCherry-Moesin/UAS-RhoV14* (Fig. 4 I), *Oregon-R* (Fig. S3 C), and *ubi-Moesin-GFP,srp-Gal4>UAS-mCherry-Moesin/+;UAS-tre1-RNAi.P{GD715}v7220/+* (Fig. S3, G–K).

### Time-lapse microscopy and wounding

White prepupae (0 h APF) were collected and maintained at either 25°C or 29°C until the required stage. For RNAi-mediated knockdown experiments, crosses were maintained at 18°C to minimize Gal4-mediated expression, and newly selected 0-h APF pupae then shifted to 25°C or 29°C to boost Gal4 activity. For imaging, pupae were dissected from their cases and mounted on glass-bottomed dishes (MatTek) using heptane glue. 75-h APF pupal wings were wounded using a nitrogen-pumped ablation laser (Spectra-Physics) attached to a Zeiss Axioplan 2 widefield imaging system. For microinjections, Cascade Blue Dextran (3,000 mol wt, final concentration of 5 mg/ml), Fluoresbrite Polychromatic Red Microspheres (1 µm diameter, 1:10 dilution), or Fluoresbrite YG Microspheres (0.5 µm diameter, 1:10 dilution) were injected into the thorax using a FemtoJet micromanipulator (Eppendorf). Imaging was performed on a Leica TCS SP5-II confocal laser-scanning microscope or light-sheet microscope (Lightsheet Z.1, Zeiss). Image preparation and analysis were performed using Volocity (PerkinElmer) and ImageJ (National Institutes of Health) software; bead velocities were quantified using the automated tracking feature within Volocity. Statistical analysis was performed using Prism software. For 3D reconstructions, imaging data were processed using IMARIS software (Bitplane). Figures were prepared using Adobe Photoshop, Adobe Illustrator, or Adobe InDesign software. Vein lumen position was determined by fluorescent dextran injection or careful examination of GFP-tagged Moesin labeling in individual z-sections.

### Analysis of hemocyte extravasation and morphology

Hemocyte extravasation duration was defined as the time taken from the initial breaching of the vessel wall to when the cell body had completely moved across the vein wall into the intervein region. To quantify hemocyte morphology during extravasation events, maximum intensity Z-projections were generated from 4D imaging data to produce a single-plane time series. All images were convolved with a Gaussian kernel (σ = 1 pixel) to remove high-frequency noise. The series was binarized using a single intensity threshold calculated for the entire stack using Huang’s algorithm (Huang and Wang, 1995). Individual contiguous regions were manually selected for analysis and skeletonized using ImageJ (Schindelin et al., 2012; Schneider et al., 2012). Branches containing fewer pixels than a user-defined threshold were removed from further analysis. The remaining skeleton was analyzed using the Analyze Skeleton ImageJ plugin (Arganda-Carreras et al., 2010).

### Immunostaining and in situ hybridization

Pupae were dissected from pupal cases and wounded, as above, before fixation for 10 min in a 1:1 mix of 8% paraformaldehyde with heptane. Pupal wings were removed and further fixed for 30 min in 4% paraformaldehyde before washing in 1× PBS/0.1% Triton-X. For immunostaining, pupal wings were blocked in 1% BSA in PBS and then incubated in primary antibody overnight at 4°C. Primary antibodies were detected using appropriate AlexaFluor488-, AlexaFluor568-, or AlexFluor647-conjugated secondary antibodies (Molecular Probes). Pupal wings were mounted on a glass slide in Vectashield (Vector Labs), and imaging was performed on a Leica SP5 confocal microscope. Immunostaining was performed with the following antibodies: anti-MMP1 (mix of 3A6B4, 3B8B12, and 5H7B11, all 1:10; Developmental Studies Hybridoma Bank), anti-GFP (1:500; Abcam), and anti-RFP (1:500; MBL). In situ hybridization was performed using standard techniques (Wilk et al., 2010), and *tre1* transcript was detected using an antisense RNA probe transcribed from the *tre1* cDNA clone RE07751 (BDGP Drosophila Genomics Resource Center).

### RNA isolation, reverse transcription, and real-time quantitative PCR

RNA was isolated from control and *srp-Gal4>UAS-tre1-RNAi(TRiP.HMS00599)* 75-h APF pupae by crushing in TRIzol (Life Technologies) and RNA purified using an RNeasy Mini Kit (Qiagen). Equal quantities of RNA were then reverse transcribed using a Thermo Scientific Maxima First Strand cDNA Synthesis Kit, and genomic DNA was eliminated using double strand-specific DNase (Thermo Scientific). Relative quantification of gene expression was performed using SYBR Green Supermix with a real-time PCR machine. *Tre1* gene expression was normalized to the expression of the housekeeping reference gene α-tubulin84B using the ΔΔCt analysis method. The following primers were used in this study: Tre1 F-primer 5′-ATTAGTGCCTGTGTCTTTGTGAC-3′ and R-primer 5′-GGAGATGCTTAGCGAAATGACG-3′; and α-tubulin84B F-primer 5′-CACACCACCCTGGAGCATTC-3′ and R-primer 5′-CCAATCAGACGGTTCAGGTTG-3′.

### CLEM

75-h APF pupae were prepared for confocal microscopy as above. At appropriate time points, pupae were transferred to a glass vial for fixation (2% paraformaldehyde and 1.5% glutaraldehyde in 0.1 M sodium cacodylate buffer 1:1 with an equal volume of heptane) for 1 h at room temperature. Samples were washed three times (0.1 M sodium cacodylate) before transfer to distilled H_2_O for removal of the cuticle and wing dissection. Wings were fixed further for 2 h (1% osmium in 0.1 M sodium cacodylate) before washing in 0.1 M sodium cacodylate and in distilled H_2_O. Samples were dehydrated (30 min in 70%, 90%, 96%, and 100% ethanol) and incubated in propylene oxide (PPO; 3 × 20 min). PPO was replaced with a 50:50 mix of PPO:epon, incubated overnight, and then evaporated off for 2 h. Wings were transferred to fresh epon (3 g TAAB 812 Resin, 2 g dodecenyl succinic anhydride, 1.25 g methyl nadic anhydride, and 0.1875 g benzyl dimethylamine) for 24 h and then embedded/polymerized at 60°C for 72 h. Sections were cut on an Ultramicrotome (Leica EM UC6) and imaged using a Tecnai 12-FEI 120-kV BioTwin Spirit Transmission Electron Microscope with a FEI Eagle 4k × 4k charge-coupled device camera.

### Online supplemental material

Fig. S1 and Videos 1, 2, 3, 4, and 5 illustrate that migratory hemocytes become restricted to wing veins during *Drosophila* pupal wing development and wing folding. Fig. S2 and Videos 6 and 7 illustrate the gross morphological and ultrastructural details of immune cell extravasation from wild-type *Drosophila* pupal wing veins to wounds. Fig. S3 and Videos 8 and 9 illustrate the effects of modulating integrin, Tre1, and Rho GTPase activity on hemocyte extravasation.

## Supplementary Material

Supplemental Materials (PDF)

Video 1

Video 2

Video 3

Video 4

Video 5

Video 6

Video 7

Video 8

Video 9
