# Supplementary material for: Drosophila immune cells extravasate from vessels to wounds using Tre1 GPCR and Rho signaling
Source: J Cell Biol. 2018 Sep 3;217(9):3045–56. doi: 10.1083/jcb.201801013 (PMC6122984; doi:10.1083/jcb.201801013)
Supplement: Supplemental Materials (PDF) [file JCB_201801013_sm.pdf]

Supplemental material

Thuma et al., https://doi.org/10.1083/jcb.201801013

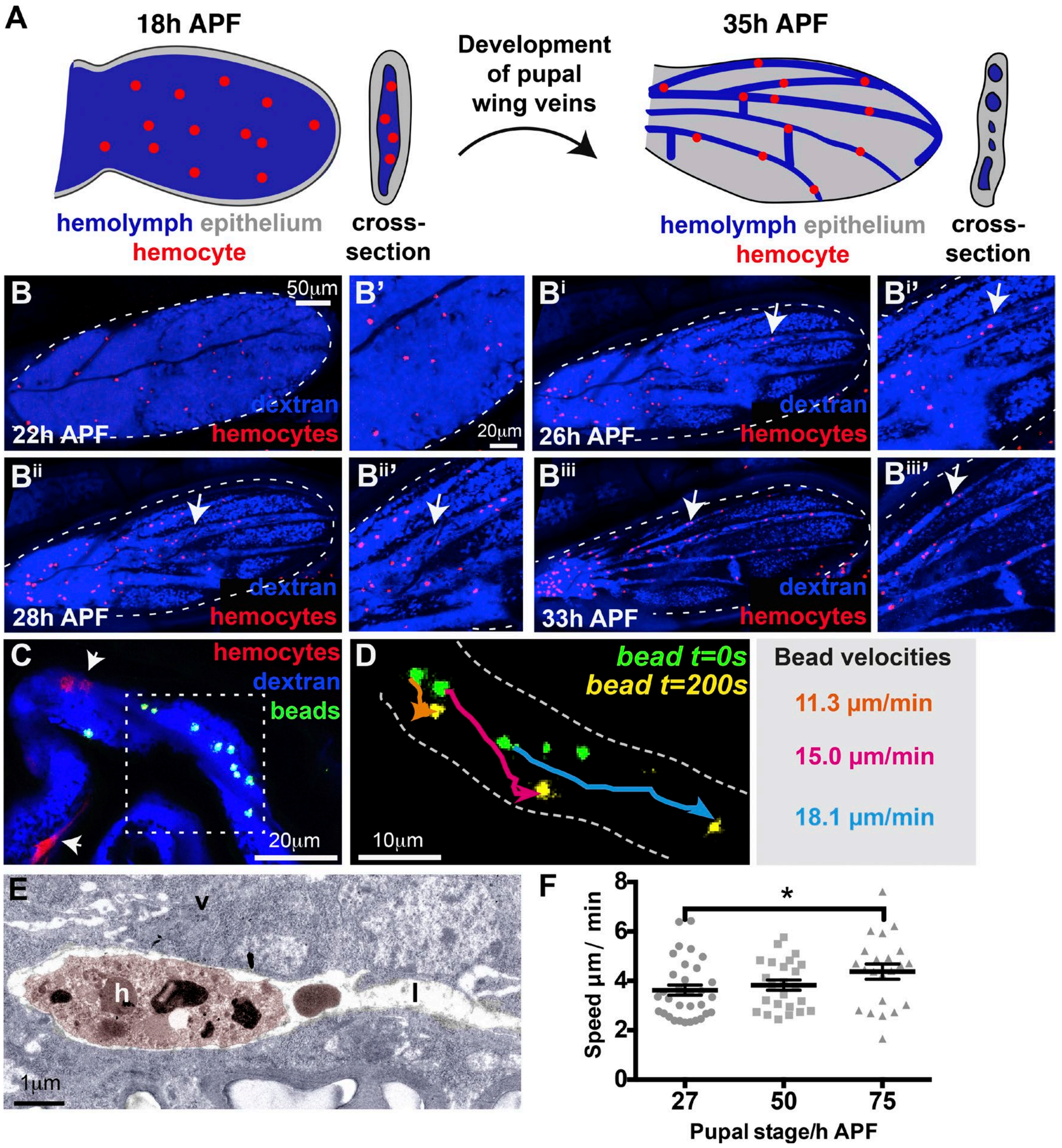

Figure S1. ***Drosophila* pupal wing hemocytes become restricted to developing wing veins that carry circulating hemolymph.** Related to Fig. 1. **(A and B)** Schematic and in vivo imaging illustrating pupal wing morphogenesis and the development of the wing veins. From 18 h to 35 h APF, the basal surfaces of the dorsal and ventral wing epithelia (gray) reorganize and adhere in the intervein regions, but the prespecified wing veins remain as open vessels. Hemolymph (shown by injected fluorescent dextran, blue), including circulating hemocytes (red, *srp-Gal4*–driven nuclear RFP), becomes progressively restricted to the wing veins (arrows, Bi–Biii). **(C and D)** Fluorescent beads (green, 1 µm diameter) injected into the wing hemolymph (labeled blue by injected dextran, C) move rapidly through the wing veins (arrowed tracks, D with bead endpoint false-colored yellow) in areas devoid of hemocytes (arrows, red, *srp>moe-mch*, C) as wing heart contractions generate a limited flow within the vessels. **(E)** TEM of hemocyte (h, false-colored pink) within the vein (v, vein walls, false-colored blue) lumen (l) of 75-h APF unwounded pupal wings (E, low-magnification view of Fig. 1 K). **(F)** Hemocyte migration speed increases from 27 h APF (before wing folding) to 75 h APF (after folding and wing heart contraction). Data are represented as mean ± SEM; *$P < 0.05$ via *t* test with Sidak's correction for multiple comparisons.

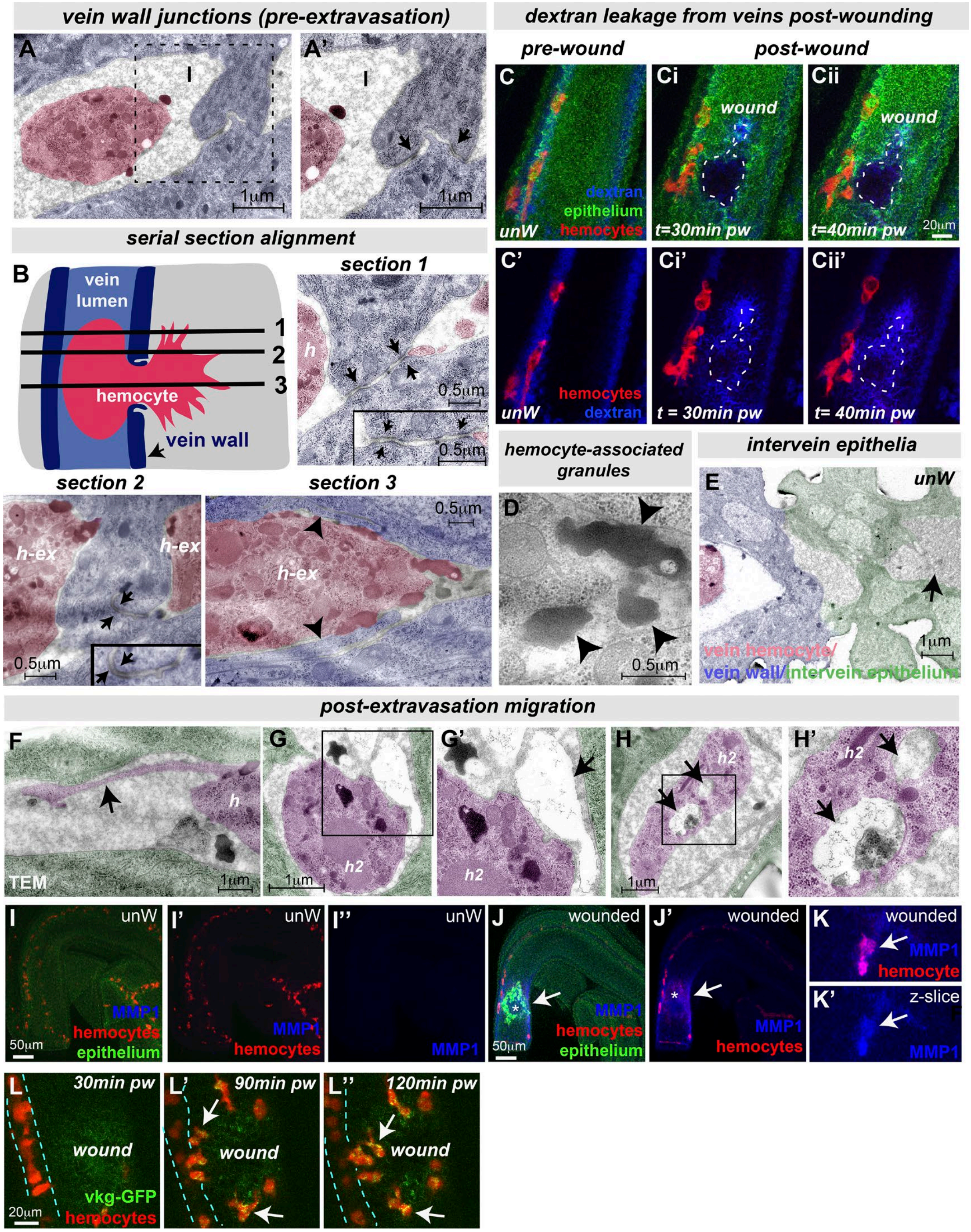

Figure S2. **Vein wall junctions are remodeled as hemocytes extravasate from vessels and migrate toward the injury site.** Related to Fig. 3. **(A and A')** Prior to extravasation, hemocytes (false-colored pink) are enclosed within the vein lumen (l) by the vein wall barrier (false-colored blue; arrows indicate vein wall junctions between opposing vein wall cells). **(B)** Analysis of serial sections through an extravasating hemocyte (schematic) reveals that the vein wall junctions remain intact (arrows, sections 1 and 2) either side of where the hemocyte cell body directly crosses the vein wall (arrowheads, section 3). h, hemocyte within lumen of vessel; h-ex, hemocyte in the process of extravasating across the vessel wall. **(C)** Prior to wounding, injected fluorescent dextran (blue) is restricted to the lumen of the pupal wing veins (C and C') that carry circulating hemocytes (red, *srp>mCherry-Moesin,* C and C'). After wounding, as hemocytes extravasate across the vessel wall (Ci and Cii), dextran leaks out from the vessel lumen into the interstitial space (Ci' and Cii'). **(D)** Extravasating hemocytes closely associated with densely staining granules at their leading edge (arrowheads). **(E)** Extracellular spaces are present between opposing intervein epithelial (false-colored green) even in unwounded pupal wings (arrow). **(F–H)** Hemocytes (false-colored magenta) that have exited the vessel display long, trailing tails (or uropods, arrow, F; hemocyte labeled h) and are associated with regions of disorganized, less dense, extracellular material (G and arrow, inset G'; hemocyte labeled h2); similar material is observed within vacuoles inside hemocytes (arrows, H and inset H'; hemocyte labeled h2). **(I–K)** MMP1 levels (blue) are elevated upon wounding (arrows, J and J') compared with unwounded pupal wings (I–I''), and single slices reveal MMP1 staining elevated within hemocytes (arrows, K and K'). **(L)** Live imaging of Collagen IV, Viking-GFP (green), reveals puncta of Collagen IV appearing within hemocytes (red, *srp>mCherry-Moesin*) following wounding (L and arrows, L' and L''). unW, unwounded; pw, post-wounding.

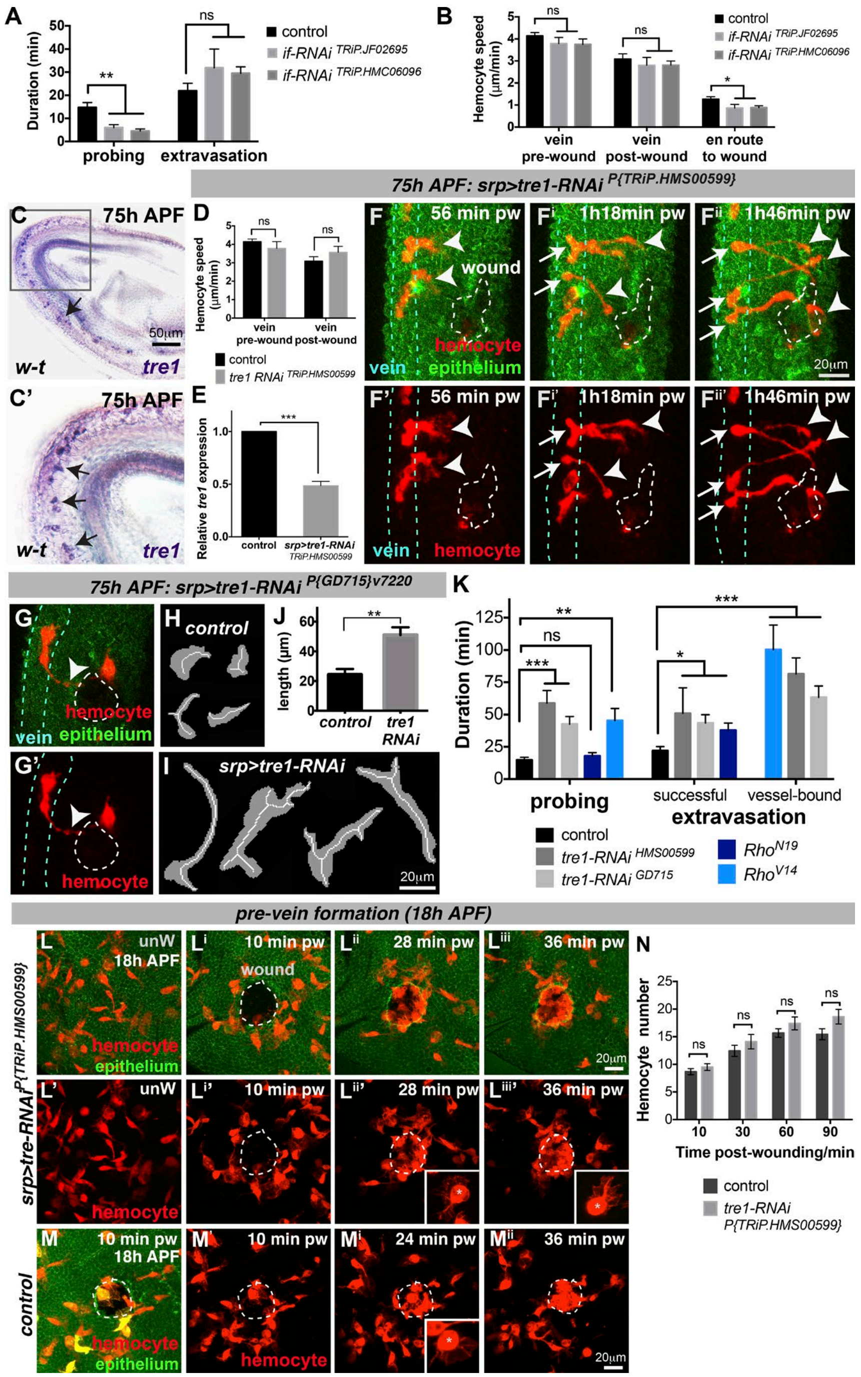

Figure S3. **The requirement for Tre1 in hemocytes is specific for wound recruitment involving transepithelial migration.** Related to Fig. 4. **(A and B)** RNAi-mediated silencing of *inflated* (using multiple RNAi lines) within pupal hemocytes (using *srp-Gal4*) causes a significant reduction in hemocyte probing of the vessel wall after wounding (A) and slows post-extravasation migration toward the wound (B), but does not affect basal hemocyte motility within the veins (B). **(C)** In situ hybridization reveals *tre1* is expressed within 75-h APF pupal wing hemocytes (arrows, C and C′). w-t, wild-type controls. **(D)** RNAi-mediated silencing of *tre1* specifically within pupal hemocytes (using *srp-Gal4*) does not affect hemocyte motility within the veins. **(E)** Quantitative RT-PCR of the relative expression of *tre1* in whole 75-h APF pupae following hemocyte-specific *tre1-RNAi*$^{P\{TRiP.HMS00599\}}$ with Tre1 threshold cycle values normalized to α-tubulin reveals a significant reduction in *tre1* expression. **(F–J)** Multiple *tre1-RNAi* lines (P{TRiP.HMS00599} in F and P{GD715}v7220 in I and J; driven in hemocytes by *srp-Gal4*) give similar defects in hemocyte extravasation; hemocytes (red, *srp>mCherry-Moesin*) become elongated and branched as they attempt to leave the vein (arrowheads, F–Fii and G; quantification in H–J), but they are unable to retract their tail ends from the vessel (arrows, Fi and Fii). **(K)** Quantification of duration of hemocyte probing and extravasation is shown for control hemocytes and hemocytes expressing *tre1-RNAi* (using P{TRiP.HMS00599} and P{GD715}v7220) and dominant-negative or constitutively active Rho (using *srp-Gal4*). **(L–N)** Inhibition of *tre1* using P{TRiP.HMS00599} RNAi in hemocytes (using *srp-Gal4*) before vein formation at 18 h APF (L–Liii) has no significant effect on hemocyte extravascular recruitment to wounds compared with controls (M–Mii, quantified in N). Asterisks indicate individual hemocytes. **(A, B, D, E, J, K, and N)** Data are represented as mean ± SEM; ns, not significant; *P < 0.05, **P < 0.01, and ***P < 0.001 via multiple *t* tests followed by Sidak's correction for multiple comparisons (A, B, K, and N) or the Mann-Whitney test (J). unW, unwounded; pw, post-wounding.

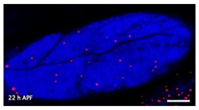

Video 1. **Hemolymph and circulating hemocytes become restricted to developing wing veins during *Drosophila* pupal development.** Related to Fig. 1. In vivo time-lapse video of the developing *Drosophila* pupal wing from 22 h APF through to 33 h APF, with time points every 5 min. As the two wing epithelia reorganize and adhere basally in the presumptive intervein regions, the hemolymph within the extracellular space (labeled by injected fluorescent dextran, blue) and the constituent hemocytes (red, labeled by *srp-Gal4*–driven nuclear RFP) relocalize to the wing veins, which remain as open vessels. The pattern of pupal wing veins established in this time resembles the mature vein pattern of adult wings. Scale bar represents 100 µm.

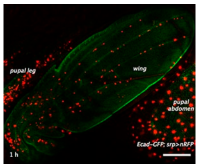

Video 2. **Pupal wings become highly folded, and veins follow a convoluted path.** Related to Fig. 1. From 45 h APF onward, pupal wings undergo dramatic expansion (two- to threefold increase in surface area) and become highly folded in a stereotypical manner within the pupal cuticle; this video shows time-lapse imaging of wing folding with time points every 8 min. Pupal wing veins remain as discrete vessels but now follow a convoluted path through the folded wings. Wing epithelium is labeled using GFP-tagged E-cadherin, and hemocytes are labeled by *srp-Gal4*–driven expression of nuclear RFP. Scale bar represents 100 µm.

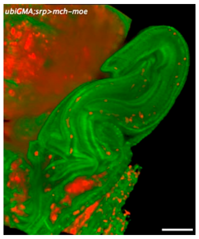

Video 3. **3D lightsheet microscopy view of vessels within the folded pupal wing.** Related to Fig. 1. In 75-h APF pupal wings, wing veins remain as discrete vessels but now follow a convoluted path through the folded wings. 3D rotations of large z-stacks generated by lightsheet microscopy indicate the highly folded nature of the pupal wings at 75 h APF, although wing veins are still visible and contain hemocytes. Wing epithelium is labeled using ubiquitously expressed GFP-tagged Moesin, and hemocytes are labeled by *srp-Gal4*–driven expression of mCherry-tagged Moesin. Scale bar represents 100 µm.

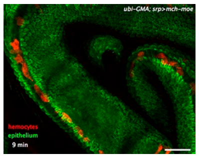

Video 4. **Hemocytes move through wing veins of 75-h APF pupae.** Related to Fig. 1. In vivo time-lapse video of the mature 75-h APF *Drosophila* pupal wing, showing hemocyte (red) movement through pupal wing veins after wing hearts have begun contractions. Time points shown every 1 min. Actin within the pupal wing epithelium is labeled using ubiquitously expressed GFP-tagged Moesin (green), and hemocytes are labeled using *srp-Gal4*–driven mCherry-tagged Moesin. Scale bar represents 50 µm.

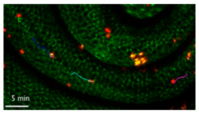

Video 5. **The relationship between hemocyte movement and hemolymph flow within wing veins of 75-h APF pupae.** Related to Fig. 1. In vivo time-lapse video of the mature 75-h APF *Drosophila* pupal wing, showing the relationship between hemolymph flow (shown by injected fluorescent beads, yellow) and hemocyte (red nuclei) movement through pupal wing vein. Tracking of bead movement (dark blue track) and hemocyte movement (green, cyan, and magenta tracks) shows hemocytes within the same vein moving both in the same (green and cyan) and opposite (magenta) direction to bead flow. Time points shown every 35 s. Actin within the pupal wing epithelium is labeled using GFP-tagged Neuroglian (green), and hemocytes are labeled using *srp-Gal4*–driven nuclear RFP. Scale bar represents 50 µm.

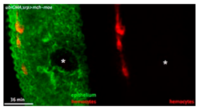

Video 6. **Hemocytes extravasate from pupal wing veins to sites of tissue damage.** Related to Fig. 2. In vivo time-lapse imaging of hemocytes extravasating from pupal wing veins of 75-h APF pupae to sites of laser-induced wounding (asterisks) in the wing epithelium (z-stack projection) with time points every 2 min. Following wounding, hemocytes probe the vessel wall before subsequently extravasating from the vessel and migrating to the site of injury; hemocytes retract their cell bodies/tails from the vein to complete transmigration and successfully exit the vessel. Wing epithelium actin is labeled with ubiquitous GFP-tagged Moesin; hemocyte actin is labeled by *srp-Gal4*–driven expression of mCherry-tagged Moesin. Scale bar represents 50 µm.

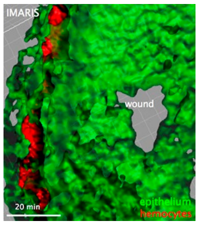

Video 7. **IMARIS view of hemocyte extravasation from pupal wing veins to sites of tissue damage.** Related to Fig. 2. In vivo time-lapse imaging of hemocytes extravasating from pupal wing veins of 75-h APF pupae to sites of laser-induced wounding (asterisks) in the wing epithelium (IMARIS visualization) with time points every 2 min. Hemocytes often return to the vessel from which they came, although other hemocytes remain at the wound site for the duration of imaging. Wing epithelium actin is labeled with ubiquitous GFP-tagged Moesin; hemocyte actin is labeled by *srp-Gal4*–driven expression of mCherry-tagged Moesin. Scale bar represents 50 µm.

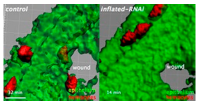

Video 8. **Hemocytes require the αPS2 integrin *inflated* for vein extravasation.** Related to Fig. 4. In vivo time-lapse imaging, with IMARIS visualization, of control (left) and *srp-Gal4*–driven *inflated-RNAi* (right) pupal wing hemocytes in response to wounding, with time points every 2 min. Unlike control hemocytes that extravasate from the wing vein toward the injury site (arrow, left), hemocytes expressing *inflated-RNAi* fail to extravasate from the vessel over 3 h of imaging (right) and continue to patrol along the vessel just as in unwounded controls. Wing epithelium actin is labeled using ubiquitously expressed GFP-tagged Moesin; hemocyte actin is labeled by *srp-Gal4*–driven expression of mCherry-tagged Moesin. Scale bar represents 25 µm.

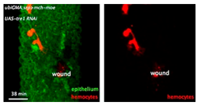

Video 9. **Hemocytes lacking Tre1 fail to extravasate from vessels to wounds and become highly elongated with defects in tail retraction.** Related to Fig. 4. In vivo imaging of pupal wing hemocytes expressing *tre1-RNAi* in response to wounding, with time points every 2 min. Unlike control hemocytes that extravasate from the wing vein toward the injury site, hemocytes with *tre1-RNAi* become significantly elongated and branched as they attempt to extravasate toward the wound, but are unable to retract their vessel-bound tails. Wing epithelium actin is labeled using ubiquitously expressed GFP-tagged Moesin; hemocyte actin is labeled by *srp-Gal4*–driven expression of mCherry-tagged Moesin. Scale bar represents 25 µm.
